# Crosstalk between DNA methylation and histone acetylation triggers GDNF high transcription in glioblastoma cells

**DOI:** 10.1186/s13148-020-00835-3

**Published:** 2020-03-17

**Authors:** Baole Zhang, Xiaohe Gu, Xiao Han, Qing Gao, Jie Liu, Tingwen Guo, Dianshuai Gao

**Affiliations:** Department of Neurobiology and Anatomy, Xuzhou Key Laboratory of Neurobiology, Jiangsu Key Laboratory of New Drug Research and Clinical Pharmacy, Xuzhou Medical University, 209 Tongshan Road, Xuzhou, 221004 Jiangsu China

**Keywords:** CREB, DNA methylation, GDNF, Glioblastoma, Histone acetylation

## Abstract

**Background:**

Glial cell line-derived neurotrophic factor (GDNF) is highly expressed in glioblastoma (GBM) and blocking its expression can inhibit the initiation and development of GBM. *GDNF* is a dual promoter gene, and the promoter II with two enhancers and two silencers plays a major role in transcription initiation. We had previously reported that histone hyperacetylation and DNA hypermethylation in GDNF promoter II region result in high transcription of GDNF in GBM cells, but the mechanism remains unclear. In this study, we investigated whether these modifications synergistically regulate high *GDNF* transcription in GBM.

**Results:**

Cyclic AMP response element binding protein (CREB) expression and phosphorylation at S133 were significantly increased in human GBM tissues and GBM cell lines (U251 and U343). In U251 GBM cells, high expressed CREB significantly enhanced *GDNF* transcription and promoter II activity. CREB regulated *GDNF* transcription via the cyclic AMP response elements (CREs) in enhancer II and silencer II of *GDNF* promoter II. However, the two CREs played opposite regulatory roles. Interestingly, hypermethylation of CRE in silencer II occurred in GBM tissues and cells which led to decreased and increased phosphorylated CREB (pCREB) binding to silencer II and enhancer II, respectively. Moreover, pCREB recruited CREB binding protein (CBP) with histone acetylase activity to the CRE of *GDNF* enhancer II, thereby increasing histone H3 acetylation and RNA polymerase II recruitment there and at the transcription start site (TSS), and promoted GDNF high transcription in U251 cells. The results indicated that high *GDNF* transcription was attributable to DNA hypermethylation in CRE of *GDNF* silencer II increasing pCREB binding to CRE in enhancer II, which enhanced CBP recruitment, histone H3 acetylation, and RNA polymerase II recruitment there and at the TSS.

**Conclusions:**

Our results demonstrate that pCREB-induced crosstalk between DNA methylation and histone acetylation at the *GDNF* promoter II enhanced GDNF high transcription, providing a new perspective for GBM treatment.

## Background

Glial cell line-derived neurotrophic factor (GDNF) is a member of the transforming growth factor (TGF-β) superfamily. The *GDNF* gene was initially cloned from the rat B49 glial cell line [1]. In human cells, *GDNF* is a single-copy gene with two promoters. Promoter I is located upstream of exon IV. Promoter II is located upstream of exon I and contains two enhancers, two silencers, and various transcription factor binding sites; it plays a major role in transcription initiation [2, 3]. Numerous studies have shown that as an important neurotrophic factor for promoting embryonic midbrain dopaminergic neuron survival and differentiation, GDNF also has nutritional and protective effects on peripheral sympathetic, parasympathetic, sensory, and motor neurons [4–6], participating in the regulation of renal cell differentiation and spermatogenesis [7–9]. GDNF has been well-known as a specific physiological neurotrophic factor and a differentiation-promoting factor for many years. However, Wiesenhofer et al. changed the understanding of GDNF when they reported abnormally increased *GDNF* expression in primary gliomas and multiple GBM cell lines, which was positively correlated with pathological grade [10]. Subsequent studies have demonstrated that abnormally increased GDNF is a powerful factor in promoting GBM cell proliferation and migration [11, 12]. Knockdown of *GDNF* or its binding receptor, *GFRα1*, effectively inhibits GBM progression [13, 14]. High GDNF concentrations protect astrocytes against DNA damage-induced apoptosis [15]. In addition, GDNF is also abnormally expressed in other malignant tumors such as pancreatic, breast, and prostate cancers, which promotes invasive tumor cell growth [16]. Abnormally high expression of GDNF is closely related to the initiation and development of malignant tumors. However, little is known about the regulatory mechanism(s). Our previous study suggested that high *GDNF* expression in GBM cells is mainly caused by its high transcription rather than gene mutations [17]. Moreover, abnormal DNA methylation and histone acetylation in *GDNF* promoter II exist in GBM tissue and cells, both of which are involved in regulating high *GDNF* transcription [18, 19]. However, it is not clear whether there is crosstalk between the two modifications and if so, what the mechanism is.

Uchida et al. recently reported that *GDNF* expression was synergistically regulated by DNA methylation and various histone modifications in different brain regions of mice [20]. Zhao et al. pointed out that the nuclear transcription factor, peroxisome proliferator-activated receptor γ was involved in the transcriptional regulation of CCAAT/enhancer binding protein α (*C/EBPα*) in bone marrow stromal cells as a cofactor for DNA methylation and histone acetylation in the *C/EBPα* promoter [21]. In addition, our previous studies indicated that abnormal epigenetic modifications in GBM cells affect the binding of multiple transcription factors to *GDNF* promoter II [17, 22]. Thus, we speculate that DNA methylation and histone acetylation may synergistically regulate high *GDNF* transcription through the abnormal binding of transcription factors. The cyclic AMP response element binding protein (CREB) is a methylation-sensitive nuclear transcription factor involved in tumor cell immortalization and transformation. It binds to the cyclic AMP response element (CRE) in the promoter as a homodimer or heterodimer and activates gene expression by binding to the KID-interacting domain (KIX) in the CREB binding protein (CBP) via its own kinase-inducible domain (KID) [23, 24]. Due to its intrinsic histone acetylase activity, CBP can efficiently acetylate histones H3 and H4 [25–27], directly stimulate the recruitment of RNA polymerase II, and loosen chromatin (especially the first nucleosome) at the transcription start site (TSS) by histone acetylation, thereby promoting eukaryotic gene expression [28]. Our previous studies revealed that hyperacetylation of histone H3 in *GDNF* promoter II in C6 GBM cells was significantly reduced by a CBP inhibitor, curcumin [19, 22, 29], suggesting that hyperacetylation may be caused by CBP. CREs are present in silencer II and enhancer II of *GDNF* promoter II, and abnormal methylation of promoter II affects the binding of CREB to promoter II in GBM tissue [17]. A recent study reported abnormally high expression and constitutive activation of CREB in glioma tissues, and latter increased with pathological grade [30]. Moreover, drug-induced phosphorylation of CREB promotes *GDNF* expression in C6 cells [31]. We hypothesized that CREB may be a coupling factor for DNA methylation and histone acetylation in *GDNF* promoter II. The abnormal DNA methylation in *GDNF* promoter II, especially hypermethylation in silencer II, results in selective CREB binding to CRE in enhancer II and increases the acetylation of histone H3 and RNA Pol II recruitment in *GDNF* promoter II by recruiting CBP, thus increasing *GDNF* transcription.

To test the above hypothesis, expression of CREB and the effect of CREB on GDNF expression were measured by real-time polymerase chain reaction (PCR), RNA interference (RNAi), and a dual-luciferase reporter system; regulation of *GDNF* transcription by the CRE in the two cis-acting elements of the *GDNF* promoter II and the methylation status and CREB binding of the two CREs were assessed by site-directed mutagenesis, CRISPR/Cas9, bisulfite sequencing PCR (BSP), and chromatin immunoprecipitation (ChIP)-PCR; effects of S133 phosphorylated CREB (pCREB) on *GDNF* transcription and recruitment of CBP were determined by point mutation, co-immunoprecipitation (co-IP), and re-ChIP-PCR; effects of CBP on histone H3 acetylation and RNA polymerase II recruitment in the CRE in enhancer II and the TSS of *GDNF* were examined by RNAi and ChIP-PCR. Our results reveal the molecular mechanisms of synergistic upregulation of *GDNF* by DNA methylation and histone acetylation in GBM cells, providing a new target for GBM treatment.

## Results

### CREB expression in GBM tissues and cell lines is significantly increased and significantly promotes *GDNF* transcription in GBM cells

**Fig. 1:**
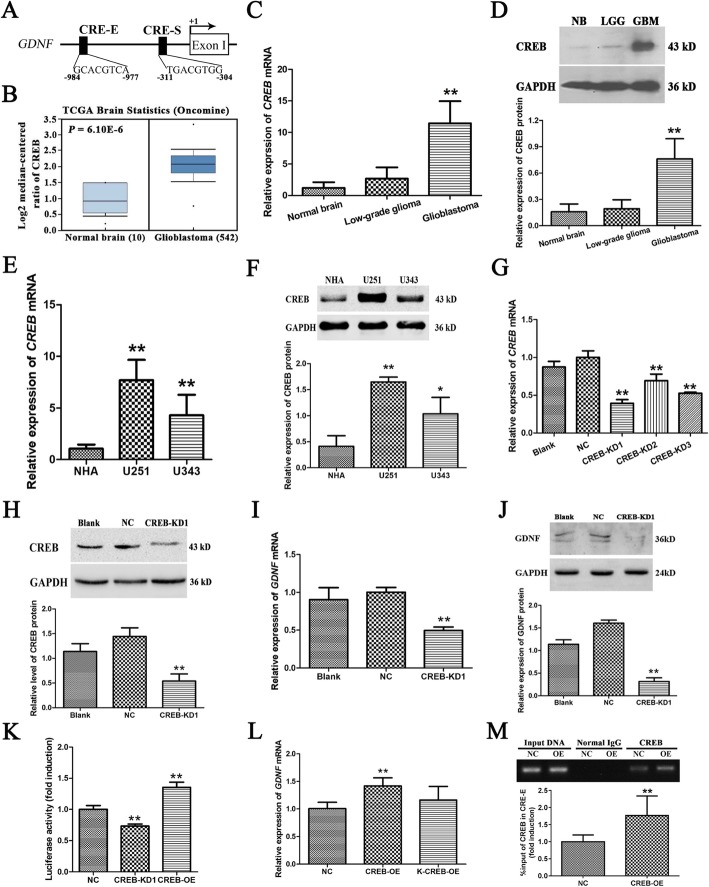
High CREB expression significantly promotes *GDNF* transcription in U251 GBM cells. **a** Putative CRE sites within the human *GDNF* promoter II. Arrows indicate the major TSS corresponding to the *GDNF* promoter II. The TSS was considered + 1. The letter E indicates enhancer II, while the letter S indicates silencer II. **b** Comparison of mRNA levels (log_2_ median-centered ratio) of human CREB in NB samples (*n* = 10) and glioblastoma brain samples (*n* = 542) from TCGA brain dataset analyzed on the Oncomine® Platform. **c***CREB* mRNA. **d** Protein expression in NB tissue and low- and high-grade GBM tissues as determined by real-time PCR and western blot (*n* = 3). **e***CREB* mRNA. **f** Protein expression in NHA cells and GBM cell lines (U251 and U343) (*n* = 3). **g***CREB* mRNA. **h** Protein expression after 72 h infection with CREB-KDs lentivirus in U251 cells (*n* = 3). **i***GDNF* mRNA. **j** Protein expression after 72 h infection with CREB-KD1 in U251 cells (*n* = 3). **k** U251 cells were transfected with the pGDNF-Luc(− 1300/+ 149)-CRE-WT and pRL-TK plasmids after 24 h of CREB-KD1 infection; and the effect of CREB expression on the *GDNF* promoter II activity was assessed by dual-luciferase assay after another 48 h of incubation (*n* = 3). **l***GDNF* mRNA expression after 72 h infection with CREB-OE and KCREB-OE in U251 cells (*n* = 3). **m** Binding of CREB to CRE in enhaner II (CRE-E) of *GDNF* promoter II after 72 h infection with CREB-OE lentivirus in U251 cells as determined by ChIP-PCR (*n* = 3). *GAPDH* was used as an internal control. All data except in (**b)** are mean ± SD. **P < 0.01

Bioinformatics analysis and Baecker et al. [3] showed a potential binding site, CRE, for the transcription factor, CREB, in both enhancer II and silencer II of *GDNF* promoter II, and the binding sites located at − 984/− 977 nt and − 311/− 304 nt, respectively (Fig. 1a). To clarify the relationship between CREB and *GDNF* transcription, CREB expression was interrogated in the Cancer Genome Atlas (TCGA) database, which revealed significantly higher *CREB* mRNA expression in GBM tissue than in normal brain (NB) tissue (*P* < 0.01) (Fig. 1b). To verify this result, *CREB* mRNA and protein expression in NB and low- and high-grade glioma (GBM) tissues were determined by real-time PCR and western blot, respectively. The results showed that *CREB* mRNA and protein expression were very significantly higher (*P* < 0.01) in GBM tissue, whereas the increase was not significant (*P* > 0.05) in low-grade glioma tissue compared with NB tissue (Fig. 1c, d). Furthermore, significantly higher *CREB* mRNA and protein expression were also observed in the human astroglioma cell lines, U251 and U343, compared to normal human astrocytes (NHA, ScienCell) (*P* < 0.05, Fig. 1e, f).

To investigate the relationship between highly expressed CREB and *GDNF* transcription, RNAi knockdown (KD) and protein overexpression (OE) of *CREB* were performed in U251 GBM cells. *CREB* mRNA expression was very significantly decreased after lentivirus CREB-KDs infection. The lowest expression was observed in the CREB-KD1 group, which was 61% lower than that of the NC group (*P* < 0.01) (Fig. 1g). A very significant decrease in CREB protein expression was also observed in this group (*P* < 0.01) (Fig. 1h). Therefore, CREB-KD1 was used for subsequent knockdown experiments. *CREB* mRNA and protein expression were very significantly increased after infection with CREB-OE lentivirus (*P* < 0.01) (Fig. S1A, B). On this basis, we found that *GDNF* mRNA and protein expression in U251 cells were very significantly decreased by *CREB* knockdown (*P* < 0.01) (Fig. 1i-j) and very significantly increased by CREB overexpression (*P* < 0.01) (Fig. S1C, D), suggesting CREB is involved in regulating *GDNF* transcription in GBM cells.

To clarify whether CREB regulates *GDNF* transcription by affecting promoter II activity, the effect of *CREB* knockdown and protein overexpression on *GDNF* promoter II activity in U251 cells was analyzed using a dual-luciferase reporter system. *GDNF* promoter II activity was significantly decreased by *CREB* knockdown and increased by CREB overexpression (both *P* < 0.01) (Fig. 1k). To determine whether CREB regulation of *GDNF* promoter II activity is dependent on its binding to promoter II, wild-type CREB and DNA-binding domain-mutated CREB (KCREB) were overexpressed in U251 cells (Fig. S2). The effect of overexpression of the two types of CREB proteins on *GDNF* transcription was assessed by real-time PCR. Overexpression of wild-type CREB significantly increased *GDNF* transcription (*P* < 0.05); whereas KCREB overexpression did not have a significant effect (*P* > 0.05) (Fig. 1l). Moreover, overexperession of wild-type CREB significantly increased the binding of CREB to CRE in enhancer II of the *GDNF* promoter II (*P* < 0.01) (Fig. 1m), suggesting CREB binding to *GDNF* promoter II is necessary for regulating *GDNF* transcription.

### CRE in the two different cis-acting elements of *GDNF* promoter II plays an opposite role in the regulation of *GDNF* transcription in GBM cells

**Fig. 2:**
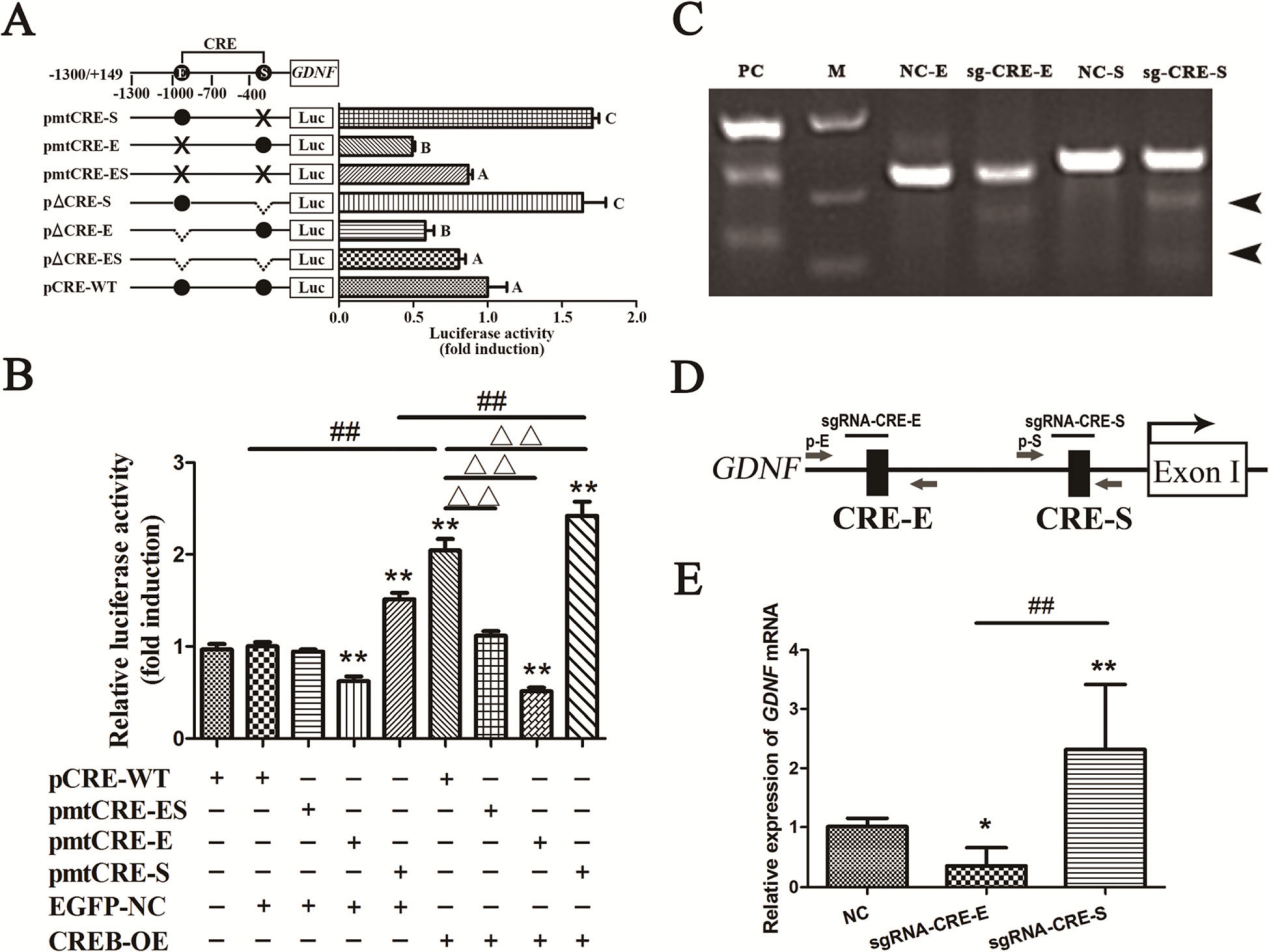
Effect of CRE in different cis-acting elements of the *GDNF* promoter II on *GDNF* transcription. **a** U251 cells in the logarithmic growth phase were seeded into 24-well plates and then transfected with 0.8 μg each of wild-type (CRE-WT), deletion (△CRE-E, △CRE-S, and △CRE-ES), or mutant plasmids (mtCRE-E, mtCRE-S, and mtCRE-ES) of the *GDNF* promoter II and 80 ng of the internal reference plasmid (pRL-TK) when the cells reached 80% confluence. Luciferase activity was assessed 48 h after transfection. Firefly luciferase activity was normalized to Renilla luciferase activity. The letter E indicates enhancer II, while the letter S indicates silencer II (*n* = 3). **b** U251 cells infected with lentivirus (CREB-OE or EGFP-NC) were treated as described in (**a)**, *GDNF* promoter II activity was assessed by dual-luciferase assay (*n* = 3). ** indicates *P* < 0.01 vs. pCRE-WT plus EGFP group; ## indicates *P* < 0.01 vs. EGFP-NC group with each type of promoter II; Δ∆ indicates *P* < 0.01 vs. wild-type *GDNF* promoter II overexpressing CREB. **c** sgRNA sites within the human *GDNF* promoter II. Thick arrows indicate the primer positions. Thin arrows indicate major TSSs. The letter E indicates enhancer II, while the letter S indicates silencer II. **d** After 72 h of infection with viral sgRNAs, the ability of the sgRNAs to direct Cas9 to cleave CRE in enhancer II and silencer II was assessed by Cruiser^TM^ assay. Black arrows indicate two DNA fragments formed by digestion. **e***GDNF* transcription in Cas9-U251 cells was determined by real-time PCR 72 h after infection with sgRNA-CRE-E or sgRNA-CRE-S (*n* = 3). All data are mean ± SD. *P < 0.05; **, ##, and different uppercase letters indicate *P* < 0.01

To determine whether CREB is involved in *GDNF* transcription through different CREs in *GDNF* promoter II, we assessed the effect of CRE deletion and mutation in different cis-acting elements on *GDNF* promoter II activity. The results showed that the activity of *GDNF* promoter II decreased slightly, but not significantly, following deletion or mutation of both CREs (*P* > 0.05). In contrast, promoter II activity was significantly increased by the deletion or mutation of CRE in silencer II (*P* < 0.01) and significantly decreased by that in enhancer II (*P* < 0.01), indicating that both mutation and deletion can block CREB binding to a specific CRE (Fig. 2a). To further clarify which binding site plays a major role in CREB regulation, we examined the effect of CREB overexpression on the activity of *GDNF* promoter II with different CRE mutations. Compared with wild-type *GDNF* promoter II plus EGFP group, promoter activity was significantly increased for the wild-type *GDNF* promoter II and that containing a CRE mutation in silencer II only and decreased for that containing CRE mutation in enhancer II only by CREB overexpression (*P* < 0.01), but it did not change significantly for that containing mutations of both CREs (*P* > 0.05). Compared with the EGFP-NC group with each type of promoter II, CREB overexpression only significantly increased promoter activity for the wild-type *GDNF* promoter II and that containing CRE mutation in silencer II only (*P* < 0.01). Compared with wild-type *GDNF* promoter II overexpressing CREB, promoter activity with CREB overexpression was significantly decreased for *GDNF* promoter II containing CRE mutation in enhancer II only (*P* < 0.01) and increased for that containing CRE mutation in silencer II only (*P* < 0.01). These findings indicate that the binding of CREB to CREs in the two cis-acting elements play opposing role in the regulation of *GDNF* transcription, and CRE in enhancer II may play a stronger role (Fig. 2b).

To corroborate the above results, we designed and packaged sgRNA lentiviruses, sgRNA-CRE-E and sgRNA-CRE-S, targeting the CRE in *GDNF* enhancer II and silencer II, respectively (Fig. 2c, Table S4). These were used to infect a U251 cell line stably expressing Cas9 protein (Cas9-U251 cells). The cleavage induced by sgRNA-CRE-E and sgRNA-CRE-S and *GDNF* transcription was then assessed by Cruiser^TM^ assay and real-time PCR. sgRNA-CRE-E and sgRNA-CRE-S were able to direct Cas9 to cleave the CRE in enhancer II and silencer II in Cas9-U251 cells (Fig. 2d). *GDNF* transcription was significantly decreased and increased by infection with sgRNA-CRE-E (*P* < 0.05) and sgRNA-CRE-S (*P* < 0.01), respectively (Fig. 2e).

### The binding of CREB to CRE in *GDNF* enhancer II is significantly increased in GBM cells

**Fig. 3:**
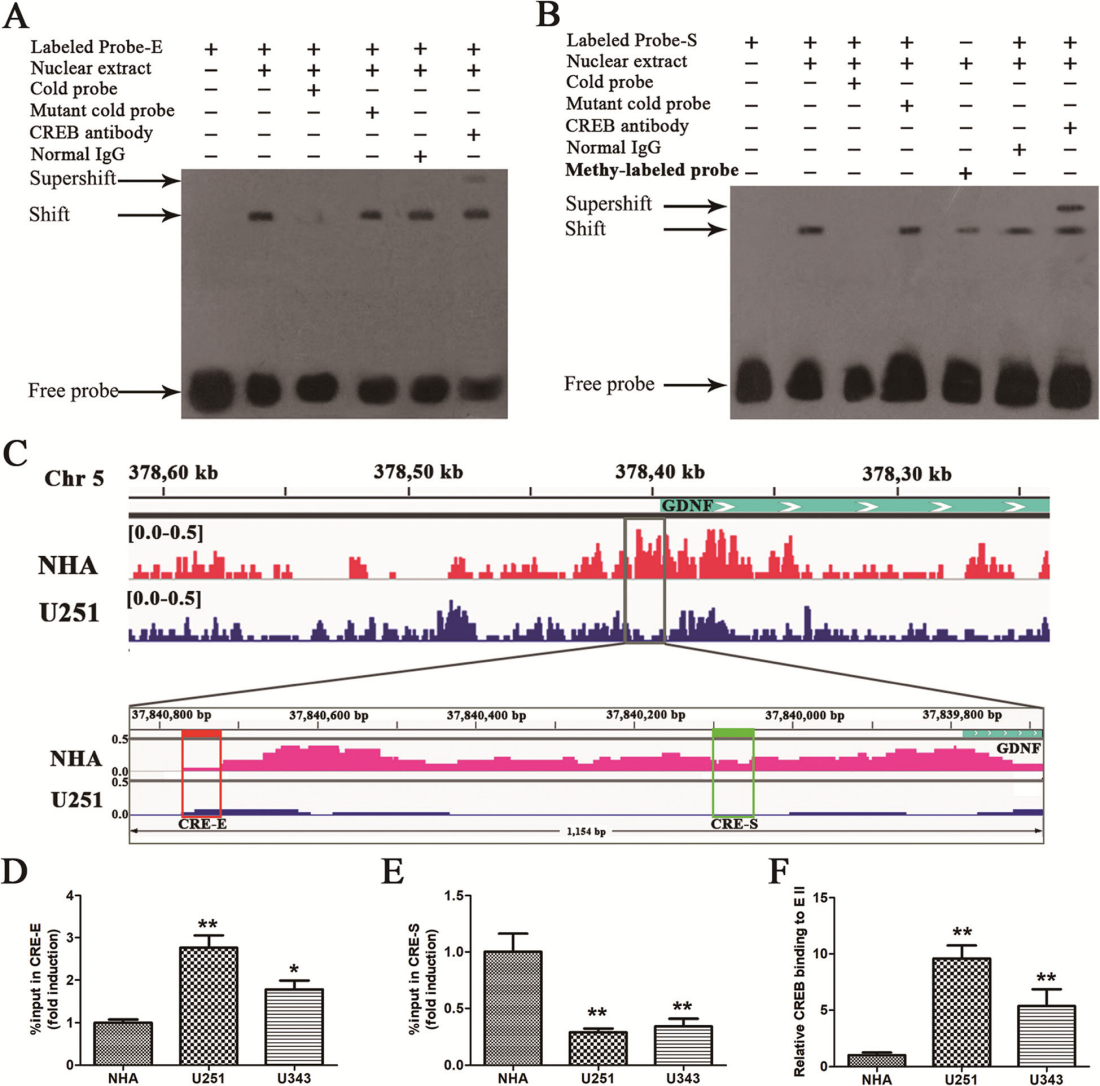
Increased CREB binding to CRE in *GDNF* enhancer II in GBM cells. **a** The binding of CREB to CRE in *GDNF* enhancer II in U251 cells as measured by in vitro EMSA. **b** The binding of CREB to CRE in *GDNF* silencer II and the effect of DNA methylation on the binding of CREB to CRE in *GDNF* silencer II in U251 cells as measured by in vitro EMSA. **c** The binding of CREB to CRE in different cis-acting elements of *GDNF* promoter II in U251 and NHA cells (*n* = 1), as assessed by ChIP-seq. The upper panel displays the peak value of CREB binding to part sequence (including the complete promoter II and part gene body of *GDNF* gene) in chromosome 5. The area demarcated with a dotted line denotes the 1154 bp fragment containing two CREs. The red and green boxes indicates the 50-bp region around CRE in enhancer II and silencer II, respectively; the peaks in the boxes indicate the binding signal of CREB to the regions; and the small blue box indicates the location of a portion of *GDNF* (*n* = 1). **d, e** The binding of CREB to CRE in different cis-acting elements of *GDNF* promoter II. **f** The relative binding to enhancer II in U251, U343, and NHA cells as determined by ChIP-PCR. *Relative binding of CREB to enhancer II = binding of CREB to enhancer II/binding of CREB to silencer II* (*n* = 3). All data except in (**c)** are mean ± SD. *P < 0.05; **P < 0.01

To determine whether CREB binds to the CRE in different cis-acting elements of *GDNF* promoter II in GBM cells, the binding of CREB to the CRE in enhancer II and silencer II in U251 cells was detected by electrophoretic mobility shift assay (EMSA). CREB specifically bound to CRE in *GDNF* enhancer II and silencer II in vitro, and hypermethylation of silencer II reduced CREB binding (Fig. 3a, b). To further investigate intracellular binding of CREB to the CRE in different cis-acting elements of *GDNF* promoter II, the binding of CREB to the CRE in enhancer II and silencer II in U251 and NHA cells was detected by ChIP-seq. The results showed that some pileup signals of CREB existed in different cis-acting elements of *GDNF* promoter, but there was no significant binding peak in U251 and NHA cells after peak calling by MACS2 (Fig. 3c). Then, ChIP-PCR was used to recheck the intracellular binding of CREB to the CRE in different cis-acting elements of *GDNF* promoter II in U251, U343, and NHA cells. The results showed that CREB bound to CRE in different cis-acting elements of *GDNF* promoter II to varying degrees; the binding of CREB to CRE in enhancer II was significantly increased (*P* < 0.05) (Fig. 3d) and that in silencer II was significantly decreased (*P* < 0.01) (Fig. 3e) in U251 and U343 cells compared with NHA cells; relative binding to enhancer II was significantly increased in U251 and U343 cells (*P* < 0.01, Fig. 3f).

### Hypermethylated CRE occurs in silencer II of *GDNF* promoter II and promotes CREB-mediated high *GDNF* transcription in GBM cells

**Fig. 4:**
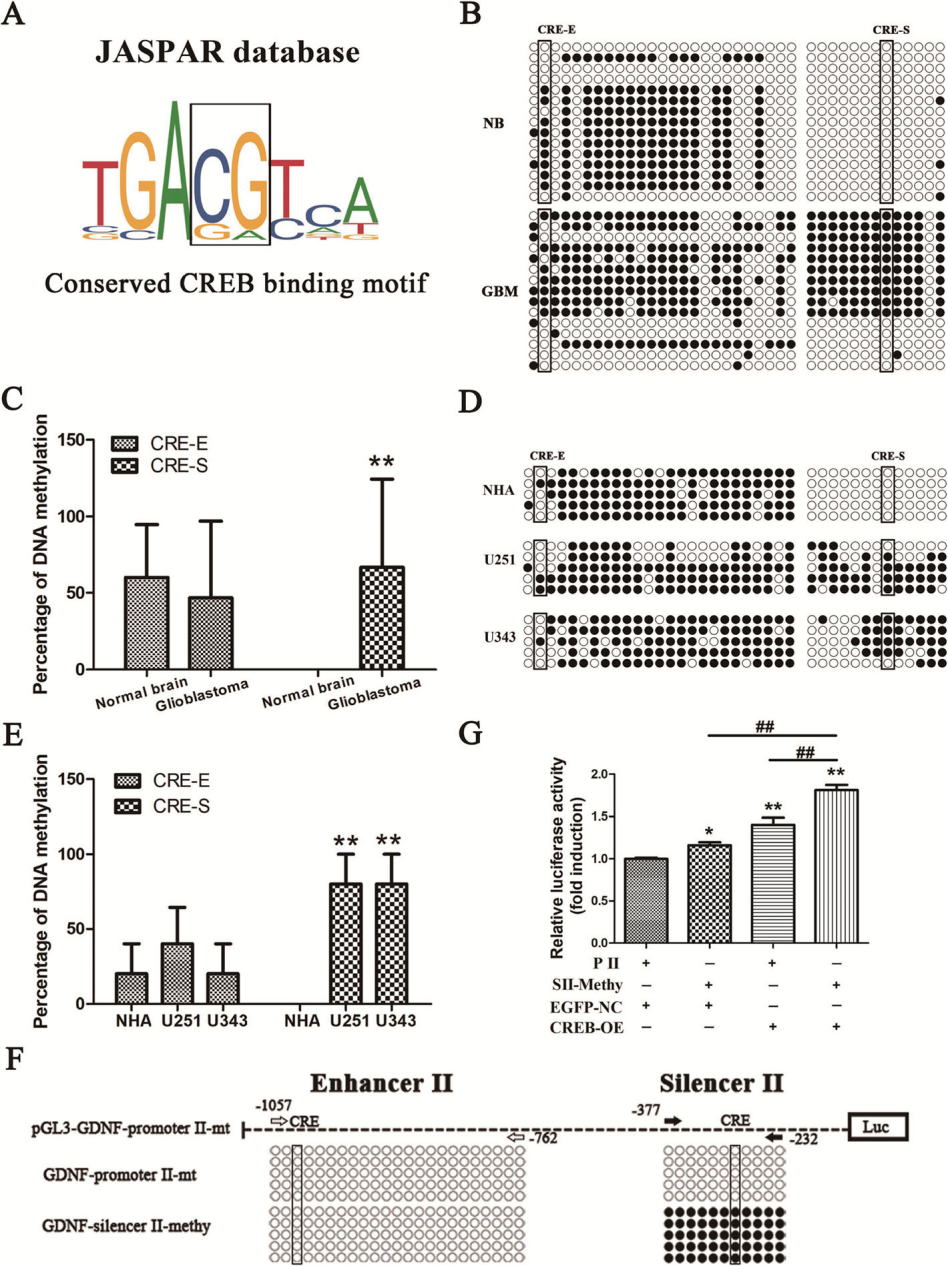
Hypermethylated CRE in silencer II of *GDNF* promoter II promotes CREB-mediated high *GDNF* transcription in GBM cells. **a** The conserved CREB binding motif (MA0018.2) in human, mouse, and rat from JASPAR database. The gray box shows a CpG dinucleotide that is a DNA methylation-sensitive site. **b** CRE methylation in different cis-acting elements of *GDNF* promoter II in GBM (*n* = 3) and NB (*n* = 3) tissues as assessed by BSP. The black box indicates CpG methylation in CRE, black circles indicate methylated sites, and white circles indicate the unmethylated sites. **c** CRE methylation levels in *GDNF* enhancer II and silencer II in GBM and NB tissues (*n* = 3). **d** CRE methylation in different cis-acting elements of *GDNF* promoter II in U251, U343, and NHA cells as assessed by BSP (*n* = 5). **e** CRE methylation levels in *GDNF* enhancer II and silencer II in U251, U343, and NHA cells (*n* = 3). **f** Circular plasmids containing *GDNF* promoter II with different methylation patterns (*n* = 5). **g** Effect of CREB overexpression on the activity of *GDNF* promoter II with different methylation patterns (*n* = 3). All data are mean ± SD. ** and ## *P* < 0.01

The conserved binding sequence of CREB is 5'-TGACGTCA-3', which contains a CpG dinucleotide that is a DNA methylation-sensitive motif (Fig. 4a). Therefore, CREB binding is sensitive to DNA methylation. We previously reported that the DNA methylation of *GDNF* enhancer II was unchanged in GBM tissue and cells, whereas hypermethylation occurred in silencer II [17, 18]. To further examine CRE methylation in different cis-acting elements of *GDNF* promoter II in GBM tissue and cells, DNA methylation of CRE in silencer II and enhancer II of *GDNF* promoter II was quantitatively assessed in GBM and NB tissues, as well as in U251, U343, and NHA cells. CRE methylation in silencer II of *GDNF* promoter II was significantly higher in GBM tissue than in NB tissue (*P* < 0.01), whereas no significant change was observed in enhancer II (*P* > 0.05, Fig. 4b, c). Similarly, CRE methylation in silencer II of *GDNF* promoter II was significantly higher in U251 and U343 cells compared to NHA cells (*P* < 0.01), whereas no significant change was observed in enhancer II (*P* > 0.05, Fig. 4d, e).

To elucidate the effect of silencer II hypermethylation on CREB-mediated regulation of *GDNF* transcription, the effect of CREB overexpression on the activity of *GDNF* promoter II in different methylation patterns was assessed by sequence-specific methylation followed by plasmid recircularization. Circular plasmids containing *GDNF* promoter II with different methylation patterns were successfully generated (Fig. 4f and Fig. S3). Hypermethylation of silencer II significantly increased *GDNF* promoter II activity (*P* < 0.05). CREB overexpression significantly increased the activity of the unmethylated promoter II and hypermethylated silencer II (*P* < 0.01). Moreover, promoter II with hypermethylated silencer II had significantly higher activity than unmethylated promoter II (Fig. 4g). This suggests that silencer II hypermethylation is involved in CREB-mediated, high *GDNF* transcription.

### pCREB recruits the histone acetylase CBP to CRE in *GDNF* enhancer II to increase *GDNF* transcription in GBM cells

**Fig. 5:**
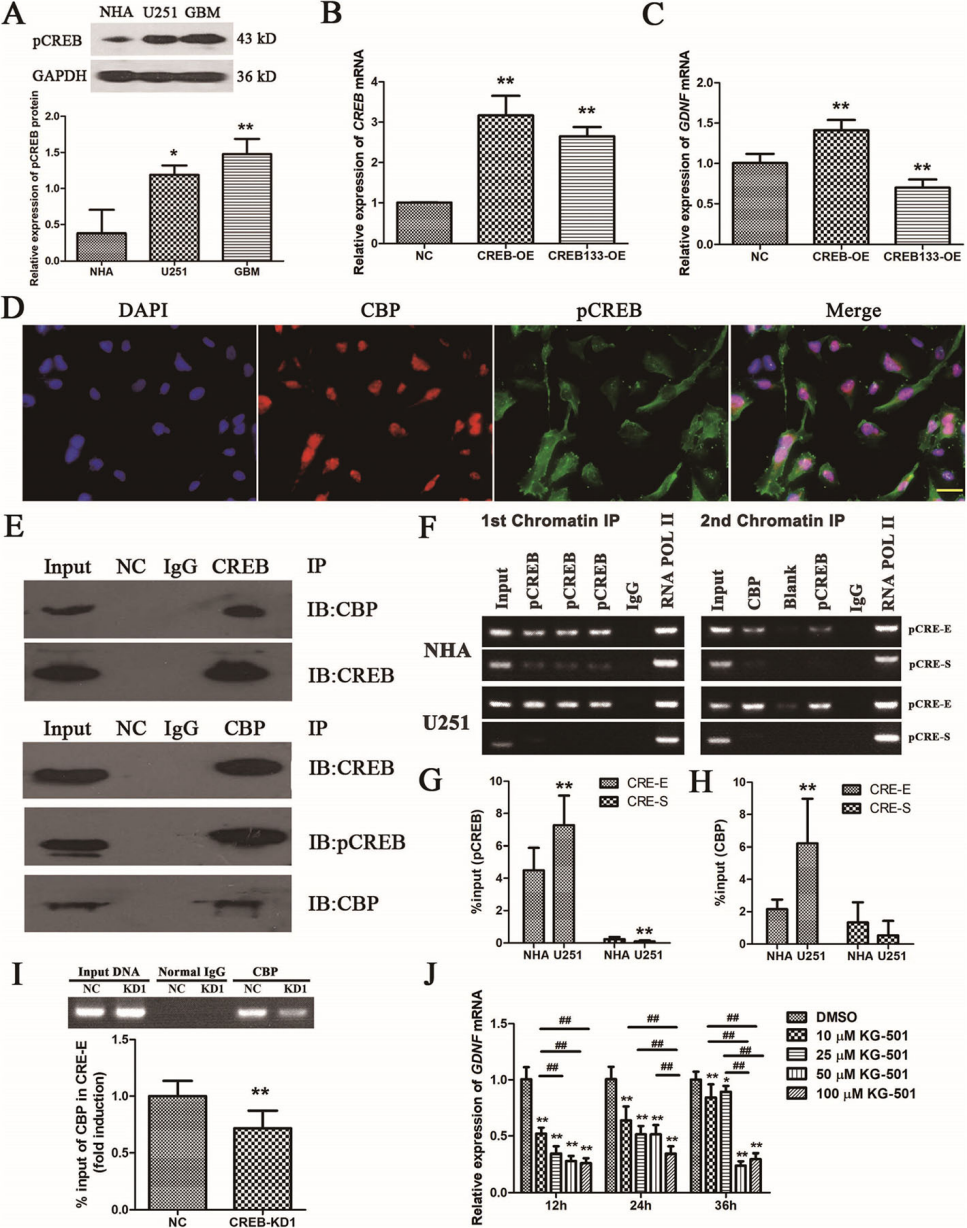
pCREB regulates high *GDNF* transcription by recruiting CBP to *GDNF* enhancer II in GBM cells. **a** CREB phosphorylation in NHA and U251 cells and GBM tissue as measured by western blot (*n* = 3). **b**, **c** Effect of overexpression of wild-type and mutant CR133 CREBs on *GDNF* transcription in U251 cells as assessed by real-time PCR (*n* = 3). **d** Immunofluorescence localization of pCREB and CBP in U251 cells. The scale is 100 μm (*n* = 1). **e** Binding of CREB/pCREB to CBP in U251 cells as measured by Co-IP. NC indicates no addition of antibodies in IP (*n* = 1). **f** Recruitment of CBP by pCREB binding to CREs in *GDNF* promoter II as determined by Re-ChIP-PCR (*n* = 1). **g** Binding of pCREB to CREs in *GDNF* promoter II as determined by the first ChIP-PCR (*n* = 3). **h** Binding of CBP recruited by pCREB to CREs in *GDNF* promoter II as determined by the second ChIP-PCR (*n* = 3). **i** Binding of CBP to CRE in *GDNF* enhancer II after knockdown of *CREB* as determined by ChIP-PCR (*n* = 3). **j***GDNF* transcription in U251 cells after KG-501 treatment as determined by real-time PCR. *GAPDH* was used as an internal control (*n* = 3). All data are mean ± SD. *P < 0.05; ** and ## *P* < 0.01

S133 phosphorylation is a key modification for CREB regulation of gene transcription [32]. A previous study found that S133 phosphorylation was abnormally increased in GBM tissue and cells [30], which is consistent with our findings (Fig. 5a). To determine whether CREB regulation of high *GDNF* transcription is dependent on S133 phosphorylation, the effect of overexpression of wild-type and S133 mutant (CR133) CREBs (Fig. 5b) on *GDNF* transcription in U251 cells was tested by real-time PCR. *GDNF* transcription was significantly increased by overexpression of wild-type CREB (*P* < 0.05) and significantly decreased by CR133 overexpression (*P* < 0.05, Fig. 5c), indicating that CREB regulation of *GDNF* transcription is dependent on S133 phosphorylation.

pCREB (S133) is capable of recruiting CBP with histone acetylase activity to promote gene transcription [33]. To determine whether pCREB promotes *GDNF* transcription by recruiting CBP, the binding of pCREB to CBP in U251 cells was assessed by immunofluorescence and co-IP. Both pCREB and CBP were present in cells and partially overlapped in the nucleus and perinuclear region (Fig. 5d), and pCREB was also able to bind to CBP (Fig. 5e). To clarify whether pCREB can recruit CBP to CRE binding sites in *GDNF* promoter II, the binding of pCREB to CREs in *GDNF* promoter II and its recruitment of CBP in U251 and NHA cells were examined by re-ChIP-PCR. pCREB mainly bound to CRE in enhancer II and recruited CBP to this site in both cell lines (Fig. 5f). pCREB binding to CRE and CBP recruitment in GDNF enhancer II was significantly increased in U251 cells compared with NHA cells (*P* < 0.01); whereas the binding of pCREB to CRE in silencer II was significantly decreased (*P* < 0.01), and CBP recruitment was decreased but not significantly (Fig. 5g, h).

To confirm that CBP is recruited to *GDNF* enhancer II by CREB, CBP binding to *GDNF* enhancer II in U251 cells infected with NC or CREB-KD1 virus was detected by ChIP-PCR. *CREB* knockdown significantly reduced CBP binding to enhancer II (*P* < 0.01, Fig. 5i). To confirm that pCREB regulation of *GDNF* transcription is dependent on CBP recruitment, U251 cells were treated with 0, 10, 25, 50, or 100 μM 2-Naphthol-AS-E phosphate (KG-501, Sigma-Aldrich) for 12, 24, or 36 h to inhibit pCREB binding to CBP. *GDNF* transcription was then measured by real-time PCR. The results showed that *GDNF* transcription was significantly and dose-dependently decreased by KG-501 treatment compared to the solvent control group (dimethyl sulfoxide) (*P* < 0.01, Fig. 5j).

### Highly recruited CBP significantly increases histone H3 acetylation and RNA polymerase II recruitment in the CRE of enhancer II and the TSS of *GDNF* in GBM cells

**Fig. 6:**
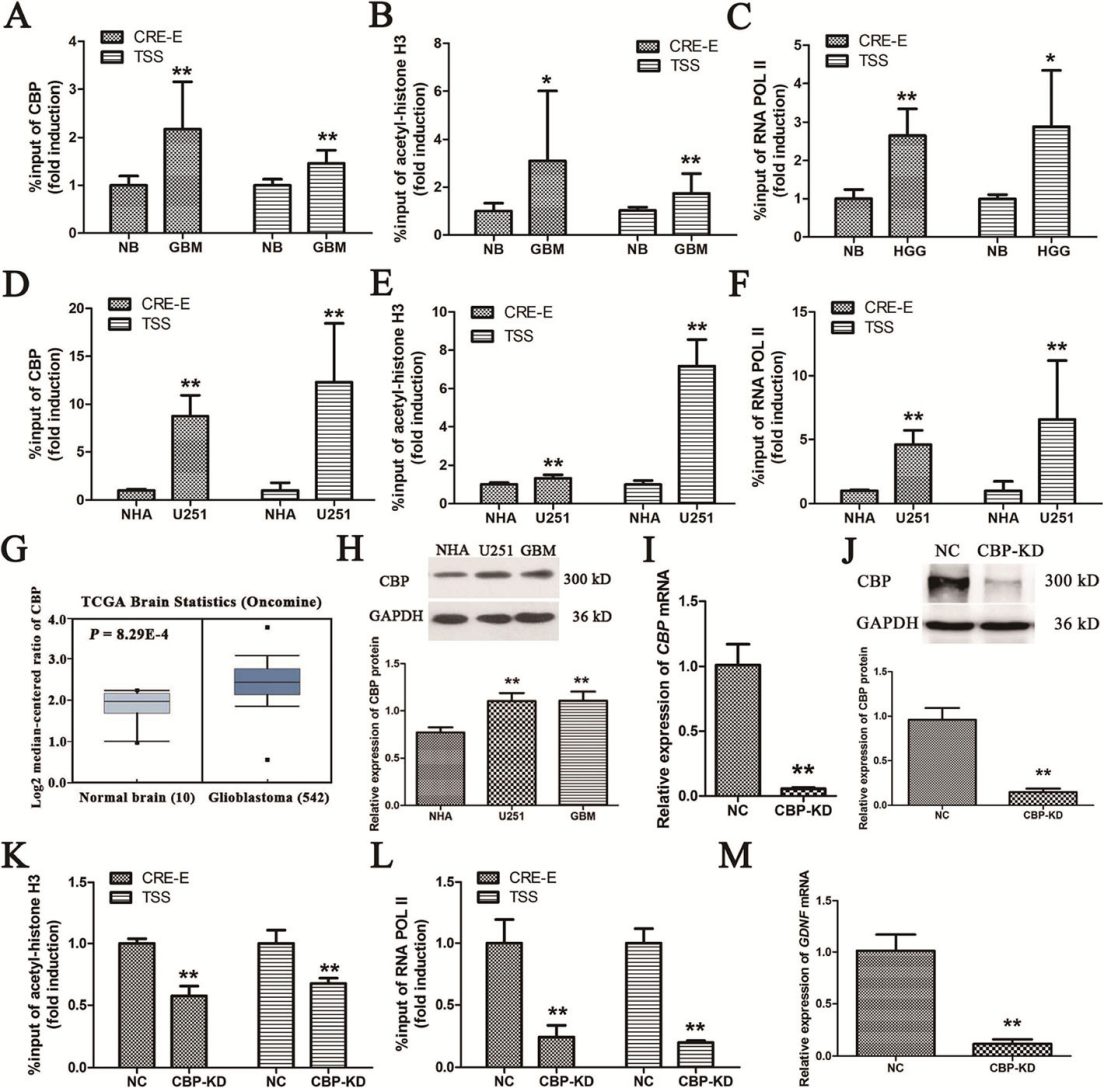
CBP significantly increases histone H3 acetylation in *GDNF* promoter II and RNA polymerase II recruitment at the TSS in GBM cells. **a** Binding of CBP to the CRE of *GDNF* enhancer II and the TSS in GBM and NB tissues (*n* = 3). **b** Histone H3 acetylation in the CRE of *GDNF* enhancer II and at the TSS in GBM and NB tissues (*n* = 3). **c** RNA polymerase II recruitment to the CRE of *GDNF* enhancer II and the TSS in GBM and NB tissues (*n* = 3). **d** Binding of CBP to the CRE of *GDNF* enhancer II and the TSS in U251 and NHA cells (*n* = 3). **e** Histone H3 acetylation in the CRE of *GDNF* enhancer II and at the TSS in U251 and NHA cells (*n* = 3). **f** RNA polymerase II recruitment to the CRE of *GDNF* enhancer II and the TSS in U251 and NHA cells (*n* = 3). **g** Comparison of mRNA levels (log_2_ median-centered ratio) of human CBP in NB samples (*n* = 10) and GBM brain samples (*n* = 542) from TCGA brain dataset analyzed on the Oncomine® Platform. **h** CBP protein expression in NHA and U251 cells and GBM tissue as determined by western blot (*n* = 3). **i***CBP* mRNA expression after *CBP* knockdown in U251 cells as determined by real-time PCR (*n* = 3). **j** CBP protein expression after *CBP* knockdown in U251 cells as determined by western blot (*n* = 3). **k** Histone H3 acetylation in the CRE of *GDNF* enhancer II and at the TSS after *CBP* knockdown in U251 cells as determined by ChIP-PCR (*n* = 3). **l** RNA polymerase II recruitment to the CRE of *GDNF* enhancer II and the TSS after *CBP* knockdown as determined by ChIP-PCR (*n* = 3). **m***GDNF* mRNA expression after *CBP* knockdown in U251 cells as determined by real-time PCR. RNA POL II indicates RNA polymerase II (*n* = 3). All data are mean ± SD. *P < 0.05; **P < 0.01

CBP possesses intrinsic histone acetylase activity [25–27] that allows it to directly stimulate RNA polymerase II recruitment and loosen chromatin (especially the first nucleosome) by histone acetylation, thereby promoting gene expression [28]. To determine whether CBP recruited to CRE in *GDNF* enhancer II has the same functions, we used ChIP-PCR to measure CBP binding, histone H3 acetylation, and RNA polymerase II recruitment in the CRE of GDNF enhancer II and at the TSS in GBM and NB tissue and in U251 and NHA cells. All three measured parameters at both sites of *GDNF* promoter II were significantly higher in GBM tissue than in NB tissue (*P* < 0.05, Fig. 6a-c). The same results were obtained in U251 cells versus NHA cells (*P* < 0.01, Fig. 6d-f).

A TCGA database search revealed significantly increased CBP expression in GBM tissue (*P* < 0.01) (Fig. 6g), which is consistent with our results (Fig. 6h). To clarify the relationship between high CBP expression and histone H3 hyperacetylation and high RNA polymerase II recruitment in *GDNF* promoter II and *GDNF* transcription, *CBP* knockdown was performed in U251 cells by infection with CBP shRNA lentiviral particles (Fig. 6i, j). ChIP-PCR revealed that *CBP* knockdown significantly reduced histone H3 acetylation (Fig. 6k) and RNA polymerase II recruitment (Fig. 6l) in the CRE of *GDNF* enhancer II and at the TSS in U251 cells (both *P* < 0.05). Real-time PCR revealed that *CBP* knockdown significantly decreased *GDNF* transcription in U251 cells (*P* < 0.01) (Fig. 6m).

## Discussion

To investigate the mechanism of crosstalk between DNA methylation and histone acetylation in regulating high *GDNF* transcription in GBM cells, we examined expression of the proto-oncogene *CREB* in different grades of glioma tissues and GBM cell lines. CREB expression was abnormally increased in GBM tissue and cell lines, which is consistent with previously reported results [30, 34]. Drug-induced CREB phosphorylation increases *GDNF* transcription in C6 GBM cells [31], suggesting that high CREB expression may be involved in regulating high *GDNF* transcription in GBM cells. Our results confirmed this hypothesis and revealed that *GDNF* expression and transcription were significantly decreased or increased by knockdown or enhancement of CREB expression, respectively. Moreover, overexpression of mutant KCREB did not significantly alter *GDNF* transcription, and overexperession of wild-type CREB significantly increased the binding of CREB to CRE in enhancer II of the *GDNF* promoter II, indicating that CREB regulation of *GDNF* transcription is dependent on its binding to promoter II.

CREB binds to DNA sequences via the CRE motif [35]. Bioinformatics analysis revealed CREs in both enhancer II and silencer II of *GDNF* promoter II [3]. To clarify whether CREB is involved in regulating *GDNF* transcription through different CREs, the effect of CRE deletion in different cis-acting elements on *GDNF* transcription was assessed using a dual-luciferase reporter system and CRISPR/Cas9. *GDNF* transcription was significantly increased and decreased by CRE deletion in silencer II and enhancer II, respectively. The CRE in enhancer II may play a larger role in regulating *GDNF* transcription. CREB binding to different CREs in *GDNF* promoter II was assessed by EMSA and ChIP-PCR, which revealed that CREB bound to CREs in both enhancer II and silencer II. The binding in enhancer II was significantly higher in U251 and U343 cells than in NHA cells, which was associated with CRE hypermethylation in silencer II. Moreover, hypermethylation of silencer II significantly enhanced the effect of CREB overexpression in promoting *GDNF* transcription. High methylation of silencer II decreases CREB binding to CRE in silencer II and increases that in enhancer II, thereby promoting *GDNF* transcription. However, why does increased binding of CREB to CRE in enhancer II increase *GDNF* transcription? Is it related to histone H3 hyperacetylation in *GDNF* promoter II? These questions need to be addressed in future studies.

CREB is activated by Ser133 phosphorylation through oncogenic signaling pathways [36]. Our study found significantly increased phosphorylation of CREB at Ser133 in GBM tissue and cells, which is consistent with the results of Valeria et al. [37]. Moreover, overexpression of S133 mutant CREB significantly reduced *GDNF* transcription. Takebayashi and Hisaoka groups showed that drug-induced phosphorylation of CREB at Ser133 increased GDNF production through extracellular signal-regulated kinase (ERK) signal pathway in rat C6 glioma cells [38–40]. In addition, Patel et al. recently reported that activated CREB by sodium benzoate significantly upregulated *GDNF* transcription in mouse astrocytes in vivo [41]. These results indicate that overactivated CREB is involved in regulating *GDNF* transcription in GBM cells. Valeria et al. found that CREB phosphorylation was only significantly increased in astrocytoma; no significant changes were seen in oligodendroglioma [37]. TCGA database analysis also revealed significantly increased *GDNF* expression in astrocytoma but no significant change in oligodendroglioma (data not shown). It suggests that pCREB may only be involved in regulating high *GDNF* transcription in astroglioma cells. Phosphorylated CREB is able to enter the nucleus and recruit coactivators by forming homodimers or heterodimers [42]. It has been suggested that phosphorylated CREB can bind to the KIX of its transcriptional coactivator, CBP, via its own KID and recruit CBP to the CRE motif of the target gene promoter, thereby activating target gene expression [33]. We therefore hypothesize that pCREB may promote high *GDNF* transcription by recruiting CBP to the CRE in *GDNF* enhancer II. To test this hypothesis, the binding of pCREB and CBP in the nuclei of GBM cells was confirmed by immunofluorescence and co-IP. A significant increase in pCREB-mediated CBP recruitment in the CRE of *GDNF* enhancer II was detected by re-ChIP-PCR. Furthermore, RNAi knockdown of *CREB* significantly reduced the binding of CBP to CRE in *GDNF* enhancer II, indicating that high recruitment of CBP in the CRE of enhancer II is achieved by pCREB. Finally, U251 cells were treated with different concentrations of KG-501, a small-molecule inhibitor that inhibits pCREB-CBP complex formation by directly targeting the KIX domain of CBP, thereby inhibiting CREB-mediated gene transcriptional activation [43, 44]. Treatment with KG-501 significantly and dose-dependently decreased *GDNF* transcription. Collectively, the results suggest that pCREB promotes *GDNF* transcription in GBM cells by increasing CBP recruitment in the CRE of enhancer II.

CBP recruited by pCREB has histone acetylase activity and can efficiently acetylate histones (H3 and H4) in mammalian cells [45, 46]. We found significantly higher CBP expression in GBM tissue and cells, which is consistent with TCGA database results. Our previous studies demonstrated hyperacetylation of histone H3 in *GDNF* promoter II in C6 cells, which could be significantly reduced by a CBP inhibitor, curcumin [19, 22, 29]. Hence, we hypothesize that CBP may be responsible for histone H3 hyperacetylation in *GDNF* promoter II. To test this hypothesis, ChIP-PCR was performed to measure CBP binding and histone H3 acetylation in the CRE of *GDNF* enhancer II in GBM and NB tissues and in U251 and NHA cells. The results showed that both parameters were significantly increased in GBM tissue and cells. Histone H3 acetylation in the CRE of *GDNF* enhancer II was significantly decreased in U251 cells by *CBP* knockdown, indicating that CBP is involved in the hyperacetylation of histone H3 in *GDNF* enhancer II. Numerous studies have shown that histone H3 hyperacetylation can open the promoter chromatin, thereby facilitating the binding of regulatory factors [47]. In addition, CBP can bind to the DNA region involving H3K36 acetylation by the bromodomain [48]. CBP phosphorylated at Y1126 can bind to acetylated histones, H3K27, H3K14, H3K9, and H3K4 [49]. Thus, it can be seen that CBP increases histone H3 acetylation in the CRE of *GDNF* enhancer II, and then promotes the binding of CBP and enhancer II through the two mechanisms indicated above, thereby forming a positive feedback pathway to maintain hyperacetylation of histone H3 in the CRE of enhancer II in GBM cells. Moreover, CBP has been shown to promote the recruitment and release of promoter-proximal RNA polymerase II by increasing histone acetylation in the first nucleosome, thereby promoting the expression of eukaryotic genes [28]. Our previous study has shown that histone H3 hyperacetylation in *GDNF* promoter II in C6 cells promotes the increase in RNA polymerase II recruitment at the TSS [29]. However, the mechanism of action is not clear. Thus, we hypothesize that CBP recruited to the CRE of *GDNF* enhancer II may act on the TSS via a loop structure, thereby increasing histone H3 acetylation and RNA polymerase II recruitment at this site. This hypothesis is confirmed by the findings that CBP binding, histone H3 acetylation, and RNA polymerase II recruitment at the TSS of *GDNF* were significantly increased in GBM tissue and cells, and that histone H3 acetylation and RNA polymerase II recruitment at this site were significantly decreased by *CBP* knockdown.

## Conclusions

**Fig. 7:**
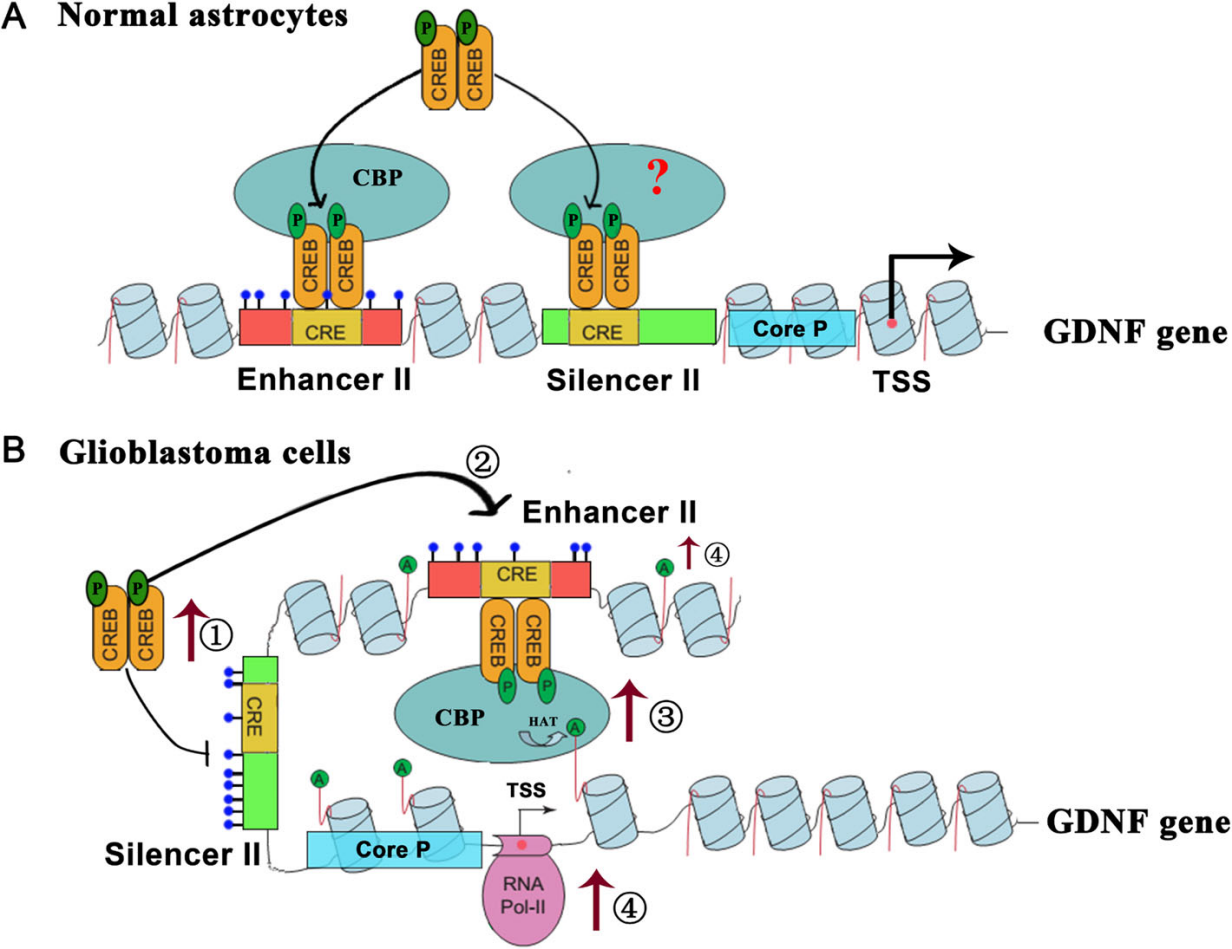
A schematic diagram of molecular mechanisms showing how CREB promotes *GDNF* transcription in GBM cells as a cofactor for DNA methylation and histone acetylation. **a** In normal astrocytes, *GDNF* silencer II is not methylated, and pCREB binds to the CRE in enhancer II and silencer II. Binding of pCREB to the CRE in silencer II is significantly higher in normal astrocytes than in GBM cells. Binding of pCREB to CRE in silencer II activates the silencing action of silencer II and reduces *GDNF* expression. **b** In GBM cells, CREB is significantly upregulated and phosphorylated. Hypermethylation of silencer II, especially the CRE, inhibits its binding to pCREB, thereby increasing the binding of pCREB to CRE in enhancer II. pCREB recruits CBP to enhancer II and the TSS, enhances histone H3 acetylation and RNA polymerase II recruitment at these sites, and ultimately leads to high *GDNF* transcription. The letters P and A stand for phosphorylation and acetylation, respectively. The bat structure represents DNA methylation. Blue cylinders represent nucleosomes. Red lines indicate histone N-termini

In summary, this study provides the first evidence that the proto-oncogene CREB promotes *GDNF* transcription in GBM cells as a coupling factor for DNA methylation and histone acetylation. The molecular mechanisms are as follows: (1) CREB is highly expressed in GBM cells and is activated by S133 phosphorylation; (2) hypermethylation of *GDNF* silencer II, especially in its CRE, inhibits pCREB binding to silencer II, which results in an increase in its selective binding to CRE in enhancer II as a homodimer; (3) CBP has histone acetylase activity, is highly expressed in GBM cells, binds to pCREB, and is recruited to the CRE of *GDNF* enhancer II; (4) CBP recruited by pCREB significantly increases histone H3 acetylation in the CRE of *GDNF* enhancer II, which facilitates CBP binding; and CBP also acts on the TSS of *GDNF* via a DNA loop structure to increase histone H3 acetylation and RNA polymerase II recruitment at this site, thereby increasing *GDNF* transcription (Fig. 7). Our future study will focus on investigating how pCREB activates silencer II to downregulate *GDNF* transcription in GBM cells or normal astrocytes.

## Methods

### Tissue samples

Normal brain (NB) tissue samples were obtained from 6 patients with acute brain trauma who underwent intracranial decompression, and 12 human glioma tissue samples (World Health Organization [WHO] grades I-IV) were acquired from the affiliated hospitals of Xuzhou University. Biopsied glioma tissues derived from patients who had not yet undergone cancer therapy were randomly sampled. Specimens with pathological grades I-II and IV were assigned to the low- and high-grade glioma (glioblastoma, GBM) groups, respectively.

### Cell culture

Human U251 and U343 GBM cell lines were obtained from ATCC between years 2015 and 2018 and cultured as described previously [18]. The cell lines were authenticated by short tandem repeat profiling by ATCC or Shanghai Genechem Co., Ltd. and passaged continuously for fewer than 6 months after receipt in our laboratory for relevant studies reported here. Normal Human Astrocytes (NHA, ScienCell, Carlsbad, CA, USA) were cultured in Astrocyte Medium (AM, ScienCell) as described previously [50].

### Oncomine database analysis

The Oncomine cancer microarray database (http://www.oncomine.org) was used to identify changes in gene expression in GBM [51]. “Brain Glioblastoma vs. Normal” was established as a filter to assess the mRNA levels of specific genes, and the human genome U133A brain microarray with 557 samples and 12,624 measured genes (TCGA Brain, no associated paper, 2013) was identified in the TCGA database. Specific genes were used as a filter to examine differences in mRNA expression between GBM and normal brain tissue.

### RNA extraction and real-time PCR

RNA extraction and real-time PCR were performed as previously described [22]. Glyceraldehyde-3-phosphate dehydrogenase (GAPDH) mRNA expression was used as the internal reference, and the relative mRNA expression levels of the target genes were calculated by relative quantification (2^−ΔΔCT^). The primer sequences for the target genes and internal reference gene are shown in Supplementary Table S1.

### Western blotting

Radioimmunoprecipitation assay lysis buffer was used to isolate the total protein. Samples from each group were subjected to western blotting as described previously [52]. Detailed information of the primary antibodies used in western blotting analysis is listed in Supplementary Table S2. GAPDH was used as the internal reference.

### Recombinant lentivirus construction and infection

Full-length human wild-type CREB ORF (NM_134442.3) and mutant CREB ORF (CREB-133 and K-CREB) fragments were amplified from the CREB dominant-negative vector set (pCMV-CREB, pCMV-CREB133, and pCMV-KCREB; Clontech, Mountain View, CA, USA) by PCR technology. CREB133 contains a serine-to-alanine mutation corresponding to amino acid 133 that blocks protein phosphorylation. KCREB contains mutations in its DNA-binding domain, preventing it from binding to CRE. Three types of CREB ORF were subcloned into lentiviral vector pLenti6.3-MCS-IRES-EGFP/V5 DEST. Three pairs of miRNA oligos targeting the human CREB mRNA (Supplementary Table S3) were synthesized and cloned into the lentiviral vector of pLenti6.3-EmGFP-MCS/V5 DEST. Two sgRNAs targeting different regions in human GDNF promoter II (AF053749) were synthesized and cloned into the lentiviral vector of pLenti6.3-MCS-IRES-EGFP/V5 DEST (Supplementary Table S4). Sequence alignment was performed to ensure correctness of the constructs. Recombinant lentiviruses were produced by 293 T cells, termed as CREB-OE, CREB133-OE, K-CREB-OE, CREB-KD1, CREB-KD2, CREB-KD3, sgRNA-CRE-E, and sgRNA-CRE-S. We used pLenti6.3-MCS-IRES-EGFP/V5 DEST or pLenti6.3-EmGFP-NC-miR as a negative control (NC). CBP (sc-29244-V) and control (sc-108080) shRNA lentiviral particles were purchased from Santa Cruz Biotechnology (Dallas, TX, USA). U251 cells without or with stably transfected Cas9 were plated at a density of 2 × 10^4^ cells/well in a 24-well plate (Corning, Corning, NY, USA). After 24 h, cells were infected by 1 μL concentrated lentivirus in the presence of polybrene (8 μg/mL).

### Cruiser^TM^ assay

Cruiser^TM^ assay was performed to check the lentivirus-sgRNA’s cleavage by using a knockout and mutation detection kit (GENESci, Shanghai, China) according to the manufacturer’s instructions. Briefly, genomic DNA was extracted from U251 cells with stably transfected Cas9 after 72 h of infection with sgRNA lentiviruses using a QIAamp DNA Mini and Blood Mini Handbook kit (Qiagen, Hilden, Germany). The products were amplified by two pairs of PCR primers (Supplementary Table S5). The amplification consisted of initial denaturation at 95 °C for 90 s; 35 cycles of denaturation at 95 °C for 30 s, annealing at 55 °C for 30 s, and extension at 72 °C for 20 s; 72 °C for 5 min; 98 °C for 3 min. The system was then allowed to cool to below 40 °C to obtain the mismatched hybrid DNA products for cleavage by Cruiser^TM^ detecase, a member of the CEL family of mismatch-specific nucleases. Positive clones were identified on 2% agarose gel electrophoresis. The size of the amplified fragment for the positive control was 456 bp, and the digested fragments were 303 and 151 bp.

### Construction and mutagenesis of the *GDNF* promoter-reporter construct

According to the human *GDNF* promoter II sequence obtained from GenBank (accession no. AF053749), the following sequences were obtained by chemical synthesis with a *KpnI* restriction site introduced into the 5'-end and a *HindIII* restriction site into the 3'-end: wild-type *GDNF* promoter II (− 1300/+ 149) sequence, CRE-WT; mutant or deletion sequences: mtCRE-E or △CRE-E, with CRE mutant or deletion in enhancer II; mtCRE-S or △CRE-S, with CRE mutant or deletion in silencer II; mtCRE-ES or △CRE-ES, with CRE mutant or deletion in both enhancer II and silencer II, respectively. The sequences were digested with *KpnI* and *HindIII* and ligated into the pGL3-basic vector (Promega, Madison, WI, USA). The mutant sequences of the constructs are listed in Supplementary Table S6. All constructed vectors were verified by DNA sequencing.

### *GDNF* promoter activity assay

*GDNF* promoter activity assays were performed as previously described [22]. Relative luciferase activity = luciferase activity of the experimental group/luciferase activity of the control group. Relative luciferase activity of the control group was defined as 1.

### Electrophoretic mobility shift assay

Electrophoretic mobility shift assay was performed with the non-radioactive EMSA kit (Viagene Biotech, Tampa, FL, USA) according to the user manual. The double-stranded DNA probes (wild-type CREB-E: 5'-AAGCCACTGGAGGGCACGTCACGGAGT G-3', − 997 to − 970 nt; wild-type CREB-S: 5'-CGCGCCGGTTGACGTGGTGTCTCGTTCG-3', − 321 to − 293 nt, underline denotes CREs; methyl-CREB-S: 5'-**C**G**C**GC**C**GGTTG A**C**GTGGTGTCT**C**GTTCG-3', − 321 to − 293 nt, all the cytosines in the CpG of the probe were methylated) containing the consecutive putative CREB-binding site was synthesized with and without a biotin label. We mixed 10 μg of cell nuclear extracts and a 100-fold molar excess of unlabeled competing probes (including the mutated CREB-E probe: 5'-AAGCCACTGGAGGGCTACACACGGAGTG-3' and the mutated CREB-S probe: 5'-CGCGCCGGTTGTACAGGTGTCTCGTTCG-3', mutated bases are underlined) and incubated at room temperature for 20 min followed by the addition of 15 μL of labeled probes (0.1 μmol) for 20 min. For the supershift assay, samples were incubated with 1 μL of an anti-CREB monoclonal antibody (#9197, cell signaling) for an additional 20 min at room temperature. Protein/DNA complexes were then separated by non-denaturing polyacrylamide gel electrophoresis on 6% gels and transferred to nitrocellulose membranes (Millipore). Protein/DNA complexes were analyzed using streptavidin–horseradish peroxidase and developed with an enhanced chemiluminescence system (Amersham Pharmacia Biotech, Little Chalfont, UK).

### ChIP-seq, ChIP-PCR, and re-ChIP-PCR

ChIP-PCR assays were performed as previously described [22]. For re-ChIP assays, the cells were cross-linked with 0.5 M di (N-succinimidyl) glutarate and 37% formaldehyde (Sigma-Aldrich, St. Louis, MO, USA). The chromatin collected after the first ChIP was treated with re-CHIP elution buffer and subjected to the second ChIP. For ChIP-seq assays, the immunoprecipitated DNA from the CREB antibody was quantified by high-throughput sequencing, and the data were analyzed using MACS2. Peak calling was calculated by MACS2 with parameter -B -SPMR -nomodel -extsize 200 -q 0.05. Q-values are calculated from *P* values using Benjamini-Hochberg procedure. Pileup signals (bedgraph files) were converted to big-wig files using bedGraphToBigWig and visualized in the Integrative Genomics Viewer software. The results for ChIP-PCR and re-ChIP-PCR were calculated as %Input: %Input = 2^(CtInput−CtChIP)^ × Input dilution factor × 100. Antibody information and primer sequences are shown in Supplementary Table S2 and Table S7, respectively.

### BSP

BSP was performed as previously described [17, 18]. Two pairs of BSP primers (see Table S8 for sequences) were designed and synthesized for the sequence around CRE in different cis-acting elements of promoter II. Each sample was analyzed for methylation using software on the website http://quma.cdb.riken.jp/.

### Specific sequence methylation followed by plasmid recircularization

This assay was performed according to the method previously reported by our group [18]. Briefly, the plasmid pGL3-*GDNF*-promoter II-mt containing the mutant *GDNF* promoter II was extracted. In this plasmid, a methylation-insensitive single restriction site (5'-*MluI*-silencer II-*XhoI*-3') was introduced into both ends of silencer II in *GDNF* promoter II (− 1300/+ 149) by point mutation, which did not affect promoter activity [18]. Next, 10 μg of pGL3-*GDNF*-promoter II-mt plasmid was digested with the restriction enzymes *MluI* and *XhoI*. The vector backbone and corresponding silencer II fragment were recovered by agarose gel electrophoresis. Part of the recovered silencer II fragment (3 μg) was methylated using CpG methyltransferase (M.SssI; New England Biolabs, Ipswich, MA, USA) according to the manufacturer’s instructions, and then ligated back into the corresponding luciferase vector backbone with T4 ligase (methylated fragment: vector backbone = 3:1). The remaining unmethylated silencer II fragment was also ligated back into the luciferase vector backbone as a control. Ligated components were separated by gel electrophoresis. Circular plasmid DNA with a high migration rate was recovered. After BSP confirmed > 90% DNA methylation of silencer II in *GDNF* promoter II treated with M.SssI, the circular plasmid DNA was transfected into U251 cells. Promoter activity was measured using a dual-luciferase reporter system.

### Immunofluorescence

The U251 cells were grown on coverslips in a 24-well plate, fixed with 4% paraformaldehyde (w/v) for 40 min, washed for 5 min with PBS three times and permeabilized with 0.5% (w/v) Triton X-100 in PBS for 15 min. The cells were then blocked for 30 min in PBS containing 10% bovine serum albumin followed by overnight incubation with the primary antibodies against pCREB and CBP diluted in PBS containing 10% FBS. After series of washings with PBS, cells were incubated for 2 h with secondary antibodies (Life, US) conjugated with fluorescein isothiocyanate or tetra methyl rhodamine isothiocyanate in dark moist environment. After several additional washing steps, the coverslips were mounted with Hydromount containing DAPI to stain the nuclei (KeyGEN BioTECH, China). The localization of pCREB and CBP protein was examined using fluorescence confocal microscope (Leica Microsystems).

### Co-immunoprecipitation and immunoblotting

Equal amounts of nuclear protein extracts prepared from U251 cells were incubated with the pCREB antibody, CBP antibody, or normal IgG (sc-2027X, SCBT) overnight at 4 °C. Then, the agarose-conjugated protein-A/G beads (SCBT) were added into the immunocomplex and the mixture was incubated at 4 °C for another 12 h. After extensive washing with ice-cold WB/IP lysis buffer with protease inhibitors, the beads were mixed with SDS loading buffer and boiled. The proteins in the supernatant were separated by SDS-PAGE and transferred to NC membranes. Blots were incubated with blocking reagent (TBST solution containing 5% non-fat milk), primary and secondary antibodies diluted in TBST, and then developed with enhanced chemiluminescence (ECL) + plusTM Western blotting system kit (Amersham). X-ray film was exposed to the membrane and then developed.

### Statistical analysis

All data were processed with SPSS 25.0 (IBM Corp. Armonk, NY, USA). Data are expressed as mean ± standard deviation (SD). Independent sample *t* tests were used to identify significant differences in mean values between two groups. One-way analysis of variance (ANOVA) and Tukey’s post hoc test were used to compare the mean values of multiple groups. *P* < 0.05 was considered statistically significant for all tests.

## Supplementary information

**Additional file 1:.** Supplementary Tables

**Additional file 2:.** Supplementary Figures

## Data Availability

All data is available from the corresponding author on reasonable request.

## Abbreviations

BSP: Bisulfite sequencing PCR
C/EBPα: CCAAT/enhancer binding protein α
CBP: CREB binding protein
ChIP: Chromatin immunoprecipitation
co-IP: Co-immunoprecipitation
CR133: S133 mutant CREBs
CRE: Cyclic AMP response element
CREB: Cyclic AMP response element binding protein
EMSA: Electrophoretic mobility shift assay
GAPDH: Glyceraldehyde-3-phosphate dehydrogenase
GBM: Glioblastoma
GDNF: Glial cell line-derived neurotrophic factor
KCREB: DNA-binding domain-mutated CREB
KD: Knockdown
KG-501: 2-Naphthol-AS-E phosphate
KID: Kinase-inducible domain
KIX: KID-interacting domain
NB: Normal brain
NHA: Normal human astrocyte
OE: Overexpression
PCR: Polymerase chain reaction
pCREB: Phosphorylated CREB
RNAi: RNA interference
TCGA: The Cancer Genome Atlas
TGF-β: Transforming growth factor-β
TSS: Transcription start site
WHO: World Health Organization
