# Supplementary material for: Crosstalk between DNA methylation and histone acetylation triggers GDNF high transcription in glioblastoma cells
Source: Clin Epigenetics. 2020 Mar 17;12:47. doi: 10.1186/s13148-020-00835-3 (PMC7079383; doi:10.1186/s13148-020-00835-3)
Supplement: Supplementary file 1 — Additional file 1:. Supplementary Tables [file 13148_2020_835_MOESM1_ESM.doc]

**Supplementary Tables**

**Table S1** Primer sequence for real-time PCR

| **Primer name** | | **Primer sequence** | **Product** | **Annealing temperature (ºC)** |
| --- | --- | --- | --- | --- |
| *CREB* | F 5’GGAGTGCCAAGGATTGAAGA3’ | | 226 bp | 59 |
|  | R:5’ GAATGGTAGTACCCGGCTGAG3’ | |  |  |
| *GDNF* | F 5’TGACAAAGTAGGGCAGGCATGT3’ | | 116 bp | 59 |
|  | R:5’ ATCCACACCTTTTAGCGGAATGC3’ | |  |  |
| *CBP* | F 5’ CAACCCCAAAAGAGCCAAACT3’ | | 198 bp | 59 |
|  | R:5’ CCTCGTAGAAGCTCCGACAGT3’ | |  |  |
| *GAPDH* | F 5’GAAGGTGAAGGTCGGAGTC3’ | | 226 bp | 59 |
|  | R:5’ GAAGATGGTGATGGGATTTC3’ | |  |  |

**Table S2 Antibody information**

| **Antibody name** | **Application in this study** | **Catalog Number** | | **Company** |
| --- | --- | --- | --- | --- |
| CREB | Western blotting, ChIP and Co-IP | #9197 | Cell Signaling | |
| phospho-CREB(Ser133) | Western blotting, ChIP, Co-IP and IF | 17-10131 | Millipore | |
| CBP | Western blotting, ChIP, Co-IP and IF | #7389 | Cell Signaling | |
| GDNF | Western blotting | ab18956 | Abcam | |
| GAPDH | Western blotting | ab8245 | Abcam | |
| acetyl-histone H3 | ChIP | 06-599 | Millipore | |
| RNA Pol II | ChIP | ab817 | Abcam | |
| IgG (rabbit) | ChIP | A7016 | Beyotime | |
| IgG (mouse) | ChIP | A7028 | Beyotime | |

**Table S3 CREB miRNA oligo sequence**

| **Oligo name** | **Oligo sequence** |
| --- | --- |
| CREB-miR1 | F1: 5'-TGCTGTACAGCTGCATCTCCACTCTGGTTTTGGCCACTGACTGA  CCAGAGTGGATGCAGCTGTA-3' |
|  | R1: 5'-CCTGTACAGCTGCATCCACTCTGGTCAGTCAGTGGCCAAAAC  CAGAGTGGAGATGCAGCTGTAC-3' |
| CREB-miR2 | F2: 5'-TGCTGATGAGCTGCTGGCATAGATACGTTTTGGCCACTGACTG  ACGTATCTATCAGCAGCTCAT-3' |
|  | R2: 5'-CCTGATGAGCTGCTGATAGATACGTCAGTCAGTGGCCAAAAC  GTATCTATGCCAGCAGCTCATC-3' |
| CREB-miR3 | F3: 5'-TGCTGTAGAATGGTAGTACCCGGCTGGTTTTGGCCACTGACTGA  CCAGCCGGGCTACCATTCTA-3' |
|  | R3: 5'-CCTGTAGAATGGTAGCCCGGCTGGTCAGTCAGTGGCCAAAACC  AGCCGGGTACTACCATTCTAC-3' |

Note: The underlined portion indicates the target site of the miRNA on CREB mRNA.

**Table S4 sgRNA sequence for CRISPR/Cas9**

| **sgRNA name** | **sgRNA sequence** |
| --- | --- |
| sgRNA-CRE-E | 5’-AGCCACTGGAGGGCACGTCA-3’ |
| sgRNA-CRE-S | 5’-ACTCCCCGCGCCGGTTGACG-3’ |

Note: The underlined part indicates the partial binding site of CREB.

**Table S5 Primer sequence for Cruiser^TM^ digestion**

| **Primer name** | **Primer sequence** | **Product** | **Digested product 1** | **Digested product 2** |
| --- | --- | --- | --- | --- |
| GDNFp-E | F 5’ TATGTGCCTGCCCCATGTCTGG 3’ | 339 bp | 234 | 105 |
|  | R:5’ CTAGCACCGAGAAGGAGAGGTG 3’ |  |  |  |
| GDNFp-S | F 5’ CGGGAACTCCAGGGCCTCCTC 3’ | 295bp | 198 | 97 |
|  | R:5’ CTCCTCTGGGCGCACTGCCTG 3’ |  |  |  |

**Table S6 CREs sequence in the wide-type and mutant GDNF Promoter-reporter**

| **Construct name** | **CRE sequence in enhancer II** | | **CRE sequence in silencer II** | |
| --- | --- | --- | --- | --- |
| pGDNF-Luc(−1300/+149)-CRE-WT (pCRE-WT ) | | 5'-GGGcacgtcaCGG-3' | | 5'-GGTtgacgtGGT-3' |
| pGDNF-Luc(−1300/+149)-△CRE-E (p△CRE-E) | | 5'-GGGcacgtcaCGG-3' | | 5'-GGTtgacgtGGT-3' |
| pGDNF-Luc(−1300/+149)-△CRE-S (p△CRE-S) | | 5'-GGGcacgtcaCGG-3' | | 5'-GGTtgacgtGGT-3' |
| pGDNF-Luc(−1300/+149)-△CRE-ES (p△CRE-ES) | | 5'-GGGcacgtcaCGG-3' | | 5'-GGTtgacgtGGT-3' |
| pGDNF-Luc(−1300/+149)-mtCRE-E (pmtCRE-E) | | 5'-GGGCTACACACGG-3' | | 5'-GGTtgacgtGGT-3' |
| pGDNF-Luc(−1300/+149)-mtCRE-E (pmtCRE-S) | | 5'-GGGcacgtcaCGG-3' | | 5'-GGTTGTACAGGT-3' |
| pGDNF-Luc(−1300/+149)-mtCRE-ES (pmtCRE-ES) | | 5'-GGGCTACACACGG-3' | | 5'-GGTTGTACAGGT-3' |

Note: Lowercase in CRE indicates deleted bases, and underline indicates mutated bases.

**Table S7** Primer sequence for ChIP-PCR

| Gene name | Primer sequence | Annealing temperature **(℃)** | Primer length (bp) |
| --- | --- | --- | --- |
| qGDNF-CRE-E | F: 5’ GAGACCGTGTTGGCCTTAGC3’  R:5’ GGAAGCCGAGTATTTGCCAG3’ | 60 | 150 |
| qGDNF-CRE-S | F:5’ GCGCCCTCATGTCTTCACG3’  R:5’ GGGAGCACGAGACTGGTTTG3’ | 60 | 115 |
| qGDNF-CRE-TSS | F:5’ CTGCTCGGACCTCGGCTT 3’  R:5’ GGCAAGAGTTCGCAATCCTG 3’ | 60 | 154 |

**Table S8 BSP** primer sequences

| **Primer name** | **Primer sequence** | **Product (bp)** | **Annealing temperature (ºC)** |
| --- | --- | --- | --- |
| GDNF-CRE-E | F: 5'-GTGTTGGTTTTAGTATGGGGA-3' | 315 | 58 |
|  | R: 5'- CTACCRATACAAAACAACRC-3' |  |  |
| GDNF-CRE-S | F: 5'-YGGGTTTTAGAAGATTAGTTTG-3' | 165 | 58 |
|  | R: 5'-CTCCTCTAAACRCACTACCT-3' |  |  |
