# Supplementary material for: Crosstalk between DNA methylation and histone acetylation triggers GDNF high transcription in glioblastoma cells
Source: Clin Epigenetics. 2020 Mar 17;12:47. doi: 10.1186/s13148-020-00835-3 (PMC7079383; doi:10.1186/s13148-020-00835-3)
Supplement: Supplementary file 2 — Additional file 2:. Supplementary Figures [file 13148_2020_835_MOESM2_ESM.doc]

**Supplementary Figures**

**A B**


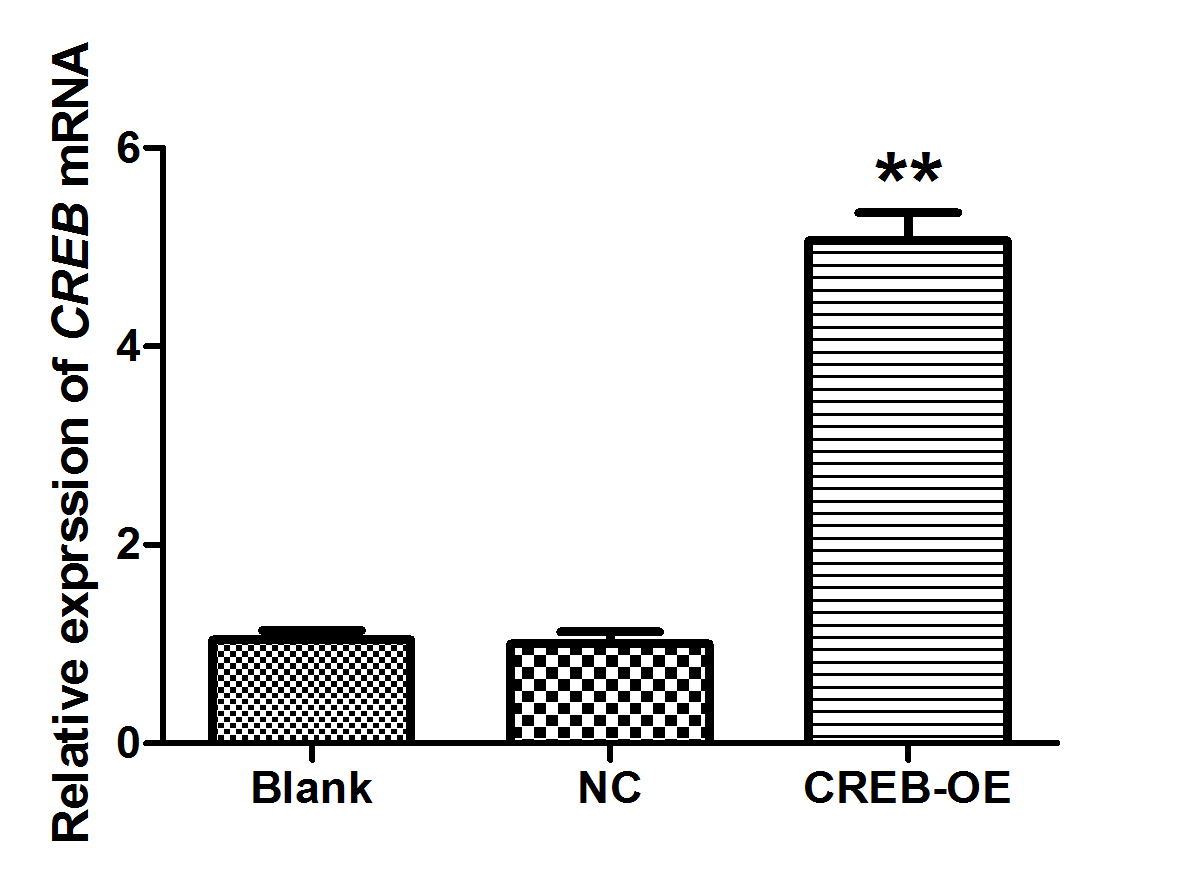

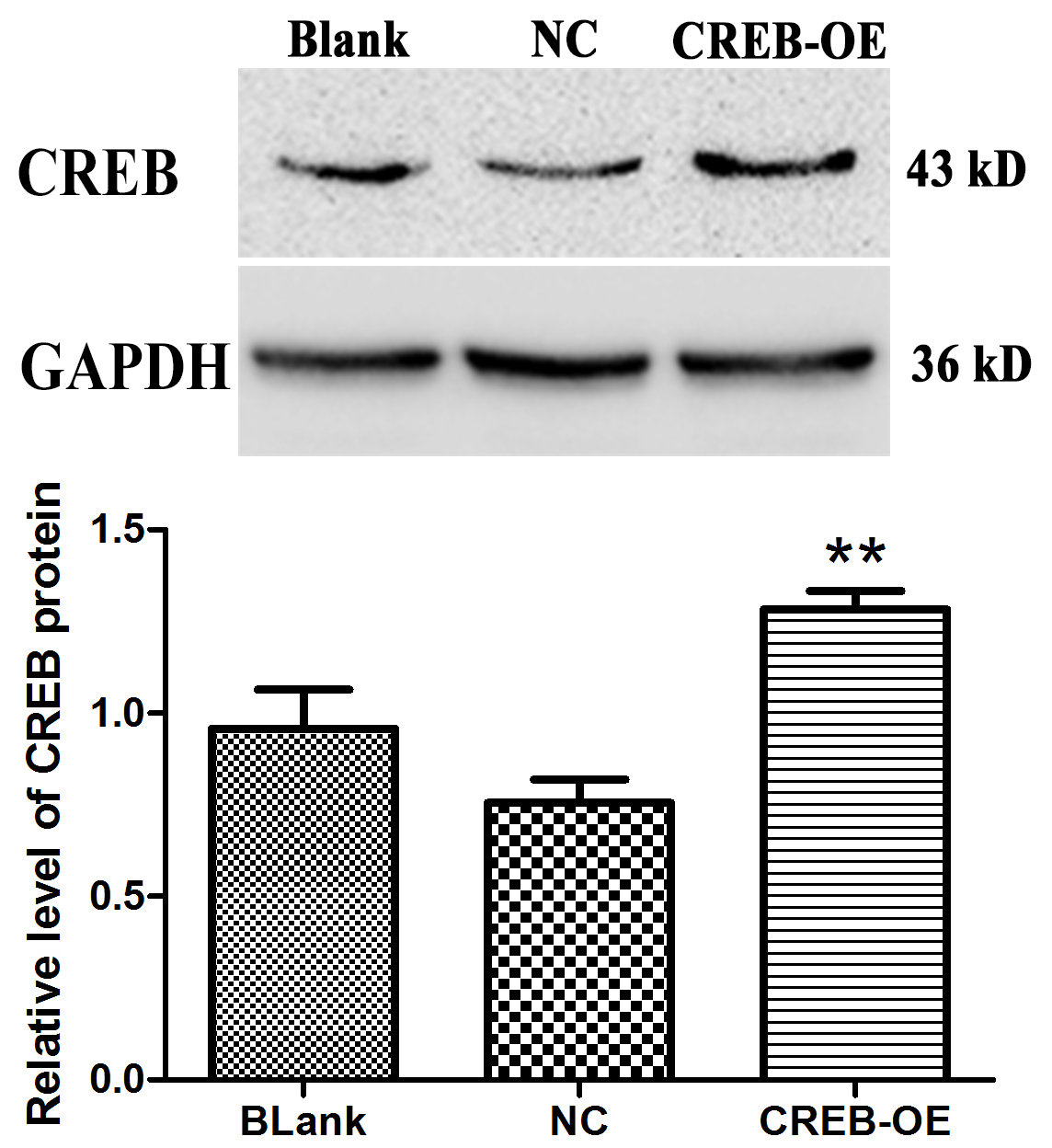


**C D**


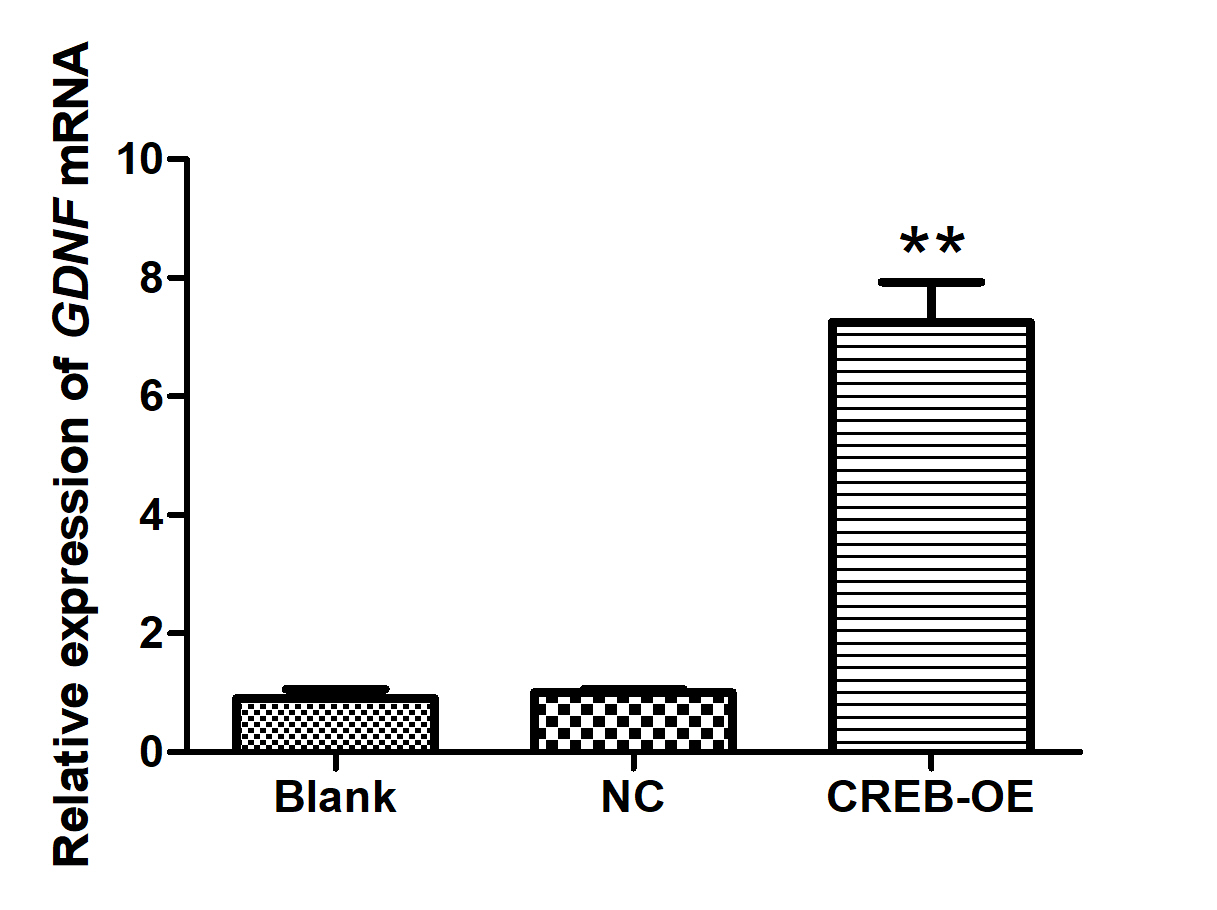
 **
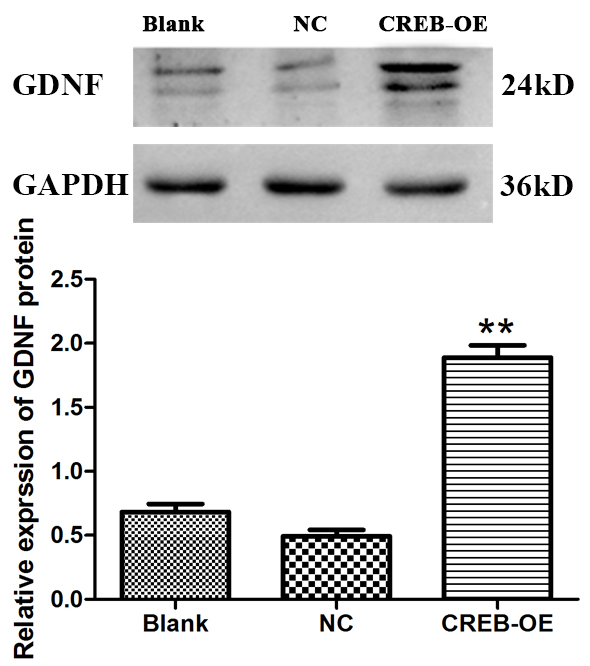
**

**Fig. S1.** CREB overexpression significantly increased *GDNF* mRNA and protein expression in U251 cells. U251 cells in the logarithmic growth phase were seeded into 12-well plates and infected with 10 multiplicity of infection of CREB-OE lentivirus when the cells reached 70% confluence. A subset of cells was used to measure mRNA and protein expression of *CREB* and *GDNF* by real-time PCR and western blot after 72 h of infection. **(a, b)** *CREB* mRNA and protein expression after infection with CREB-OE lentivirus in U251 cells. (n=3) **(c, d)** *GDNF* mRNA and protein expression after infection with CREB-OE in U251 cells. (n=3) *GAPDH* was used as an internal control. ***P* < 0.01.

**
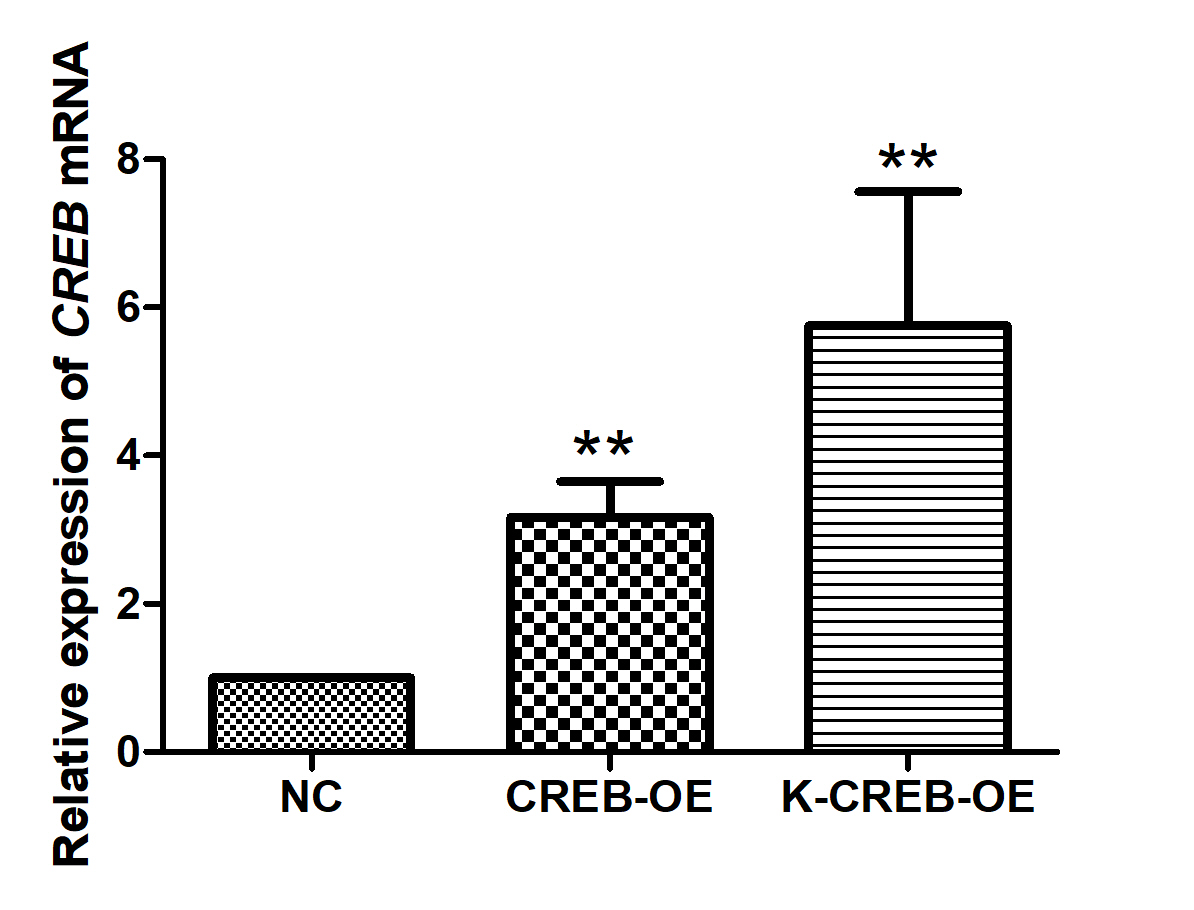
**

**Fig. S2** CREB-OE and K-CREB-OE significantly increased the expression of wild-type CREB and mutant KCREB in U251 cells. U251 cells in the logarithmic growth phase were seeded into 12-well plates and infected with 10 multiplicity of infection of CREB-OE and K-CREB-OE lentivirus when the cells reached 70% confluence. The cells were used to measure mRNA expression of *CREB* and *GDNF* (Fig. 1L) by real-time PCR after 72 h of infection. (n=3) *GAPDH* was used as an internal control. ***P* < 0.01.


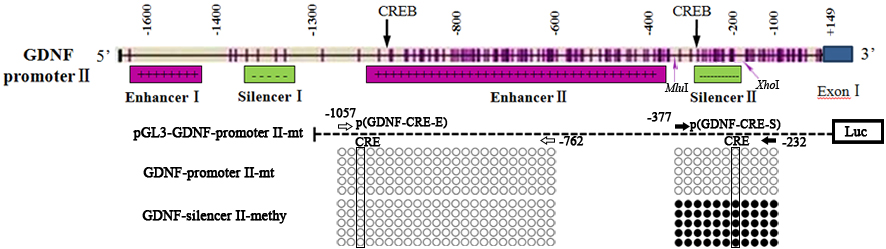


**Fig. S3** Structure of human *GDNF* promoter II. The upper part shows the relative positions of two enhancers, two silencers, CREB binding sites, and the introduced methylation-insensitive single restriction site (according to data from the EMBL-bank). Marks on the DNA scale line indicate CpG dinucleotides, and the dashed box indicates the region of interest (-1300/+149 bp). The TSS corresponding to promoter II is set to +1. Each line represents all CGs in one clone of the sample. Black circles indicate methylated sites, and white circles indicate the unmethylated sites. Black and white arrows indicate the positions of the BSP primers used, p(GDNF-CRE-E) and p(GDNF-CRE-S), respectively.
